# Newcastle Disease Virus V Protein Promotes Viral Replication in HeLa Cells through the Activation of MEK/ERK Signaling

**DOI:** 10.3390/v10090489

**Published:** 2018-09-12

**Authors:** Zhili Chu, Jiangang Ma, Caiying Wang, Kejia Lu, Xiaoqin Li, Haijin Liu, Xinglong Wang, Sa Xiao, Zengqi Yang

**Affiliations:** College of Veterinary Medicine, Northwest A & F University, Yangling 712100, China; zhilichu@126.com (Z.C.); 15193194055@163.com (J.M.); blingblingwcy@163.com (C.W.); lkj_1103@163.com (K.L.); L18584890329@163.com (X.L.); liuhai132jin@163.com (H.L.); wxlong@nwsuaf.edu.cn (X.W.); saxiao@nwafu.edu.cn (S.X.)

**Keywords:** NDV, V protein, MEK/ERK pathway, p-ERK1/2, antivirals, viral replication, infection

## Abstract

Newcastle disease virus (NDV) can infect a wide range of domestic and wild bird species. The non-structural V protein of NDV plays an important role in antagonizing innate host defenses to facilitate viral replication. However, there is a lack of knowledge related to the mechanisms through which the V protein regulates viral replication. The extracellular signal-regulated kinase (ERK) signaling pathway in the host is involved in a variety of functions and is activated by several stimuli, including viral replication. In this study, we show that both the lentogenic strain, La Sota, and the velogenic strain, F48E9, of NDV activate the mitogen-activated protein kinase (MEK)/ERK signaling pathway. The pharmacological inhibition of ERK1/2 phosphorylation using the highly selective inhibitors U0126 and SCH772984 resulted in the reduced levels of NDV RNA in cells and virus titers in the cell supernatant, which established an important role for the MEK/ERK signaling pathway in NDV replication. Moreover, the overexpression of the V protein in HeLa cells increased the phosphorylation of ERK1/2 and induced the transcriptional changes in the genes downstream of the MEK/ERK signaling pathway. Taken together, our results demonstrate that the V protein is involved in the ERK signaling pathway-mediated promotion of NDV replication and thus, can be investigated as a potential antiviral target.

## 1. Introduction

Newcastle disease virus (NDV) is a member of the genus Avulavirus in the Paramyxoviridae family. NDV infections cause a highly contagious and fatal viral disease that commonly affects most species of birds [1]. It is an enveloped virus with a negative-strand RNA genome that encodes at least six viral proteins [2]. Similar to other paramyxoviruses, NDV encodes non-structural proteins, which are named V and W. These two non-structural proteins are produced by RNA editing during the transcription of the *P* gene [3]. The V protein can block the activation of the interferon pathway in the host to facilitate NDV replication [4,5]. Through reverse genetics, Park et al. [6] showed that a recombinant NDV mutant lacking V, but with the influenza virus *NS1* gene inserted, was more infectious compared with the wild-type NDV in human cells [6]. That study suggested that the V protein plays an important role in restricting the host range and also showed that V expression delayed the apoptosis in NDV-infected chick embryo fibroblasts, but not in human HepG2 cells. Another interesting study showed that NS1 could potentially have anti-interferon and possibly anti-apoptotic effects using NS1-expressing NDV-infected cancer cells. These effects helped to prolong the survival of cancer cells and allowed the virus to replicate freely in the absence of an antiviral response [7]. Although these studies indicate that the V protein may have multiple functions, the mechanisms by which V regulates viral replication are not clear.

Extracellular-regulated protein kinase (ERK)1/2 is activated by mitogen-activated protein kinase (MEK)1/2 through the phosphorylation of threonine-183 and tyrosine-185 within its activation loop [8]. The activation of the MEK/ERK pathway is required for the normal replication of some viruses [9,10,11,12,13,14]. The herpes simplex virus 1 and dengue virus serotype 2 suppress ERK activity [15], while a suppressed ERK pathway can protect again hepatitis C viruses (HCV) through the upregulation of interferon-α [16]. Therefore, MEK/ERK may be a potential target to control NDV replication. In the case of NDV, the upregulation of the Raf kinase inhibitory protein restricts NDV replication, while this also repressed the activation of the ERK and NF-κB signaling pathways [17]. However, there are no explicit studies focusing on the relationship between ERK activity and NDV replication.

The relationship between viral replication and the ERK1/2 pathway has been elucidated for several viruses. The HCV non-structural NS5A protein is a pleiotropic phosphoprotein that mediates the ERK signaling pathway and regulates infected hepatocytes [18]. The HCV core protein can also induce cell proliferation and activate ERK [19]. Influenza A virus hemagglutinin triggers the nuclear export of the viral genome via protein kinase Cα-mediated activation of ERK signaling. This may represent an autoregulatory mechanism, which coordinates the timing of ribonucleoprotein export to ensure that all viral components are ready for viral budding [20]. Finally, the viral glycoprotein gE mediates pseudorabies virus-induced activation of ERK1/2 in different cells, which benefits viral replication [21]. However, no relationship with ERK signaling has been shown for any NDV protein.

## 2. Materials and Methods

### 2.1. Main Materials

The virus used in this study was described previously [22]. The V protein of NDV was cloned into the pCAGEN plasmid. A Flag tag was added at the multiple cloning site to create pCAGEN-Flag-V. The information related to the regents and primers is shown in Table 1.

### 2.2. Main Experimental Steps

Protocols concerning the chicken embryo use for virus production were approved by the Institutional Animal Care and Use Committee at Northwest A&F University. Cell culture, transfection, virus seeding and viral plaque analyses were conducted as described previously [22].

HeLa cells were grown to 80% confluence, before being used for transfections in 6-well plates. Plasmids (2 or 4 µg) were diluted in 400 μL of serum-free Opti-MEM with 8 μL of Lipofectamine 2000 (Thermo Scientific, Rockford, IL, USA) according to the manufacturer’s instructions. Infection with NDV utilized 0.1 or 1 multiplicity of infection (MOI) for 12 h. Inhibitor treatments were used at concentrations of 0.5–100 nM for 12 h.

For Western blotting, the total protein was extracted from treated cells. Equal amounts of protein (25 μg) were separated by 15% sodium dodecyl sulfate polyacrylamide gel electrophoresis and transferred to a nitrocellulose membrane (EMD Millipore, Darmstadt, Germany). The membrane was blocked for 12 h at 4 °C in phosphate-buffered saline containing 10% skim milk, which was followed by incubation with primary and subsequently secondary antibodies (Table 1). Detection was performed by incubating the membrane with Clarity Western ECL Substrate (Bio-Rad, Hercules, CA, USA).

For immunofluorescence analysis, cells were fixed in 4% formaldehyde for 15 min. After this, cell membranes were permeabilized with 0.5% Triton X-100 for 15 min, which was followed by blocking with 1% bovine serum albumin for 30 min at room temperature. Specific primary (overnight incubation at 4 °C) and secondary (room temperature for 90 min) antibodies were used (Table 1). Nuclei were stained with Hoechst 33342. Every step was followed by three 5 min phosphate-buffered saline washes.

For the quantitative reverse transcription polymerase chain reaction (qRT-PCR), the total RNA was extracted from cells using TRIzol reagent (Takara Bio, Dalian, China). Reverse transcription to produce cDNA was carried out using 2× PCR Mix (GeneStar, Beijing, China) and the PrimeScript RT reagent kit (Takara) on 1 µg of RNA according to the manufacturer’s instructions. The thermocycling protocol was 94 °C for 5 min, which was followed by 30 cycles of 94 °C for 30 s, 60 °C for 30 s and 72 °C for 1 min. The final extension step was 72 °C for 10 min. Amplified RNA was visualized with ethidium bromide staining after agarose electrophoresis. β-Actin was used as an internal control to normalize the relative expression of each gene.

### 2.3. Statistical Analysis

Student’s *t*-test was used when only two groups of data were compared. All data are presented as the means and SD, while the statistical significance of differences is reported as follows: * *p* < 0.05; ** *p* < 0.01; and *** *p* < 0.001. All data are representative of no less than three different experiments and the data were analyzed using GraphPad Prism 5 software (GraphPad Software, Inc., San Diego, CA, USA).

## 3. Results

### 3.1. NDV Replication Activates the ERK1/2 Pathway in HeLa Cells

**Figure 1:**
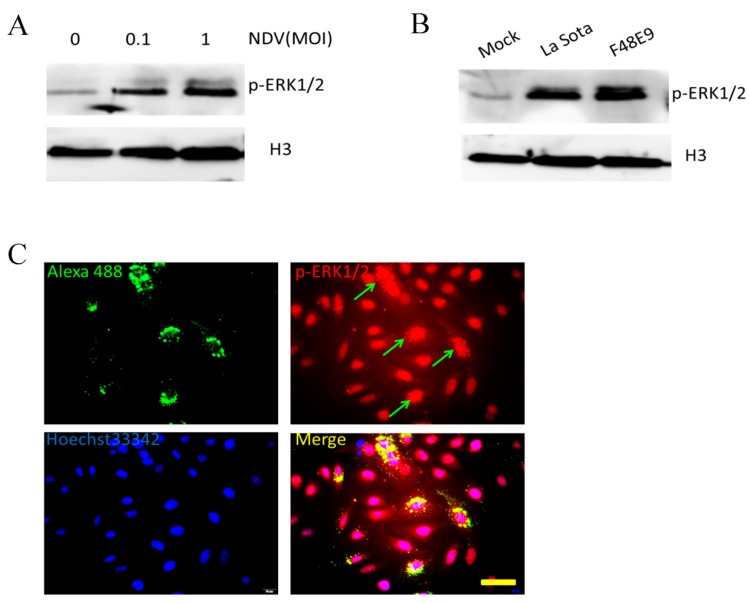
Newcastle disease virus (NDV)-induced phosphorylation of extracellular signal-regulated kinase (ERK)1/2 in HeLa cells. Phospho (p)-ERK1/2 levels in HeLa cells after (**A**) different doses of NDV (F48E9 strain; 1 multiplicity of infection (MOI)) and (**B**) different strains of NDV (La Sota and F48E9; 1 MOI). Cell lysates were analyzed 12 h post-infection by Western blotting using a p-ERK1/2 antibody and H3 histone as the reference. (**C**) HeLa cells were infected with NDV (0.1 MOI). At 12 h post-infection, the cells were stained with chicken anti-NDV serum and a rabbit anti-p-ERK1/2 antibody, which was followed by staining with goat anti-rabbit Alexa Fluor^®^ 594 (red) and goat anti-chicken IgY Alexa Fluor^®^ 488 (green) as secondary antibodies. Nuclei were subsequently stained with Hoechst 33342. Images were captured using a Leica fluorescence microscopy (400×), bar = 50 μm.

To examine whether the ERK1/2 pathway was affected by NDV in HeLa cells, we used different doses of NDV (F48E9). Twelve hours post-infection, Western blotting showed a dose-dependent increase in the levels of phospho (p)-ERK1/2 (Figure 1A). Using the NDV MTH-68/H strain, there was no correlation between IC50 values and the expression of p-ERK in different tumor cells [23]. To understand whether differences in the NDV strains affected the levels of p-ERK1/2, we performed experiments with another NDV strain (La Sota). The results showed that both the La Sota and F48E9 stains (1 MOI) activated the ERK1/2 pathway and there was no significant difference between them when using equivalent virus doses (Figure 1B). We concluded that the ERK1/2 pathway can be activated independently of the presence of NDV strain in HeLa cells.

p-ERK1/2 proteins can be located in both the cytoplasm and nucleus. Our immunofluorescence results suggested that NDV-infected cells had more p-ERK1/2 (red fluorescence) than the control group, while the additional p-ERK1/2 was mostly located in the cytoplasm (Figure 1C).

### 3.2. U0126 and SCH772984 Inhibit NDV Replication in HeLa Cells

**Figure 2:**
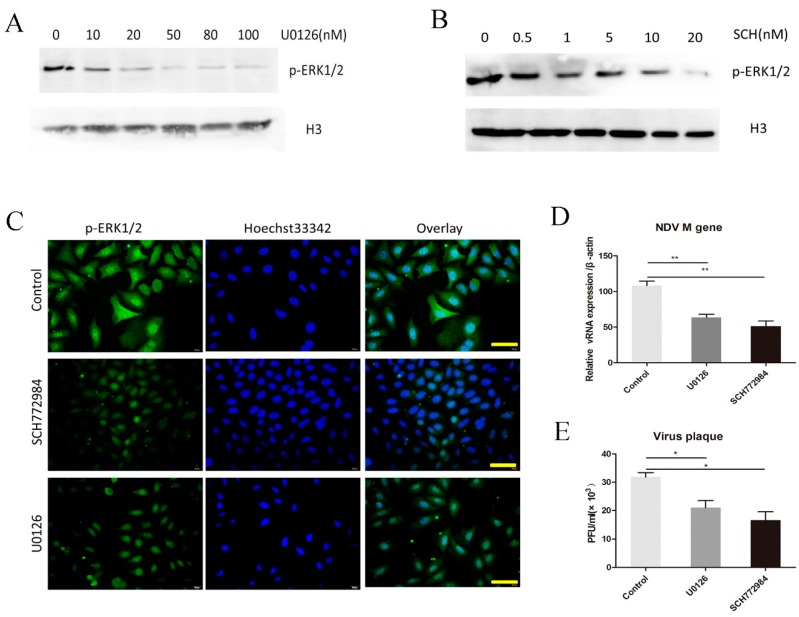
The extracellular signal-regulated kinase (ERK)1/2 inhibitors, U0126 and SCH772984, reduce NDV replication in HeLa cells. Cells were treated with various concentrations of (**A**) U0126 and (**B**) SCH772984. Lysates from treated cells were analyzed by Western blotting with the p-ERK1/2 antibody and H3 histone as a reference. (**C**) After treatment with 10 µM of the ERIK1/2 inhibitors, the expression of p-ERK1/2 protein in HeLa cells was detected using immunofluorescence (400×), bar = 50 μm. (**D**) HeLa cells were infected with NDV F48E9 (1 MOI) for 24 h and the quantitative reverse transcription polymerase chain reaction was used to measure viral RNA in cell lysates in control cells and cells treated with either 100 nM U0126 or 10 nM SCH772984, while (**E**) viral plaque formation was assessed to measure the number of viral particles in the supernatants. Data are presented as means ± SD of three independent experiments. * *p* < 0.05; and ** *p* < 0.01.

We used two ERK1/2 inhibitors, U0126 and SCH772984, to further examine the role of the ERK1/2 pathway in NDV infections. The latter inhibitor has a unique mechanism of action as it inhibits both the enzymatic activity of ERK and its phosphorylation by MEK [24,25]. The inhibitor concentrations used were 0–100 nM of U0126 and 0–20 nM of SCH772984. After treating cells for 12 h, Western blots showed that both U0126 (Figure 2A) and SCH772984 (Figure 2B) caused a concentration-dependent inhibition of ERK 1/2 phosphorylation. Immunofluorescence results showed reduced p-ERK1/2 (red fluorescence) after treatment with 100 nM U0126 or 10 nM SCH772984 for 24 h compared with the control group (Figure 2C).

To examine whether the phosphorylation of ERK1/2 affected NDV replication, we pretreated the cells with inhibitors for 12 h and infected them with NDV (1MOI), before testing the inhibitor-treated HeLa cells 24 h post-infection by measuring the viral RNA with the qRT-PCR. The results showed that in the inhibitor-treated cells, there was a 50% reduction in the virus *M* gene expression compared to the control group (Figure 2D). Viral plaque assays showed that the virus titer in the supernatant of inhibitor-treated cells was less than that in the control group (Figure 2E). Similar results have been seen for influenza A virus [26], but not for HCV [27]. The work presented here suggests that the dephosphorylation of ERK1/2 inhibits NDV replication. Conversely, we infer that the phosphorylation of ERK1/2 increases NDV replication.

### 3.3. V protein Promotes Phosphorylation of ERK1/2 and Increases NDV Replication in HeLa Cells

**Figure 3:**
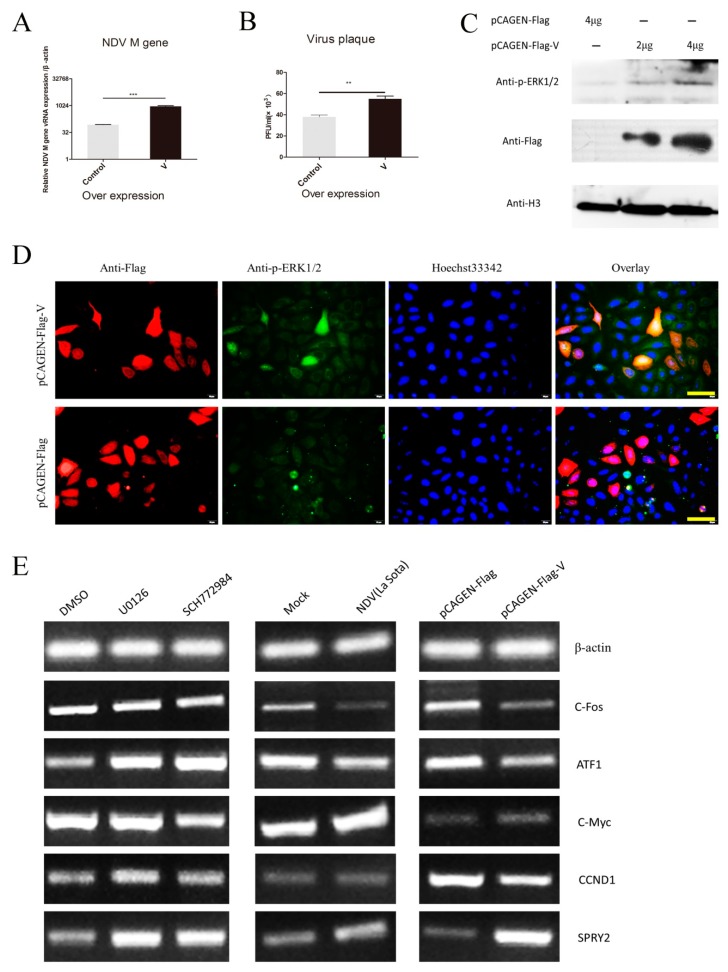
The V protein activates the extracellular signal-regulated kinase (ERK) pathway and benefits Newcastle disease virus (NDV) replication. (**A**) HeLa cells were transfected with pCAGEN-Flag (control) or pCAGEN-Flag-V (to overexpress the V protein). After 24 h, cells were infected with NDV (F48E9, 1 multiplicity of infection). After 24 h, the quantitative reverse transcription polymerase chain reaction (qRT-PCR) was used to measure viral RNA in cell lysates. Data are expressed as means ± SD of three independent experiments. (**B**) Viral plaque formation was assessed to measure the number of viral particles in supernatants. Data are expressed as means ± SD of three independent experiments. ** *p* < 0.01 and *** *p* < 0.001. (**C**) Cells were transfected with pCAGEN-Flag or pCAGEN-Flag-V. After 24 h, cells were harvested and analyzed by Western blotting with H3 histone used as a reference. (**D**) To localize the V protein and phospho (p)-ERK1/2 proteins, HeLa cells were stained with mouse anti-Flag and rabbit anti-p-ERK1/2 antibodies. This was followed by staining with goat anti-mouse IgG Alexa Fluor^®^ 594 (red) and goat anti-rabbit IgG Alexa Fluor^®^ 488 (green) as secondary antibodies. Images were captured using a Leica fluorescence microscopy (400×), bar = 50 μm. (**E**) qRT-PCR was used to analyze the expression of ERK pathway genes 24 h after transfection with pCAGEN-Flag-V, 12 h after infection with NDV or after 12 h treatments with inhibitors.

The nonstructural V protein facilitates NDV replication [4,5]. Here, we overexpressed the V protein in HeLa cells and validated this using qRT-PCR (Figure 3A). A viral plaque assay showed that the viral titer in the supernatant had similar trends to our qRT-PCR results (Figure 3B). These results suggest that the overexpression of the V protein increases viral replication in HeLa cells.

After this, we determined whether the V protein operated through the MEK/ERK pathway to regulate viral replication. We first tested the phosphorylation of ERK1/2 after overexpressing V for 24 h. Western blot results showed that compared with the control group, the p-ERK1/2 level was increased (Figure 3C). Immunofluorescence results showed that the V protein-overexpressing cells (marked with an arrow) had more p-ERK1/2 (red fluorescence) than the control group, with the added fluorescence mostly located in the cytoplasm (Figure 3D).

The key regulators of cell proliferation and tumorigenesis that are located downstream of the MEK/ERK pathway include c-fos, Elk1, c-myc and cyclin D1. The upstream regulators of the MEK/ERK pathway include SPRY2, which is an inhibitor of mitogen-activated protein kinase (MAPK) signaling. The knockdown of SPRY2 increases the phosphorylation of ERK1/2 [28]. We determined the mRNA levels of these molecules after treatment with ERK1/2 inhibitors, infection with NDV and overexpression of the V protein (Figure 3E). The results showed that NDV and overexpression of the V protein induced similar trends (up/down-regulated) in the expression of these genes. This suggests that the V protein and NDV infection activate the ERK pathway though a similar mechanism or that the V protein is the crucial factor involved in the activation of the ERK pathway by NDV. However, the activation of ERK signaling will lead to changes in downstream gene transcription. Furthermore, V protein- and NDV infection-activated ERK signaling can induce similar transcription changes in downstream genes. However, when cells are treated with ERK1/2 inhibitors, it is possible that the downstream gene changes induced by V protein overexpression/NDV infection may be blocked. ERK downstream genes, such as *c-fos*, *ATF1*, *c-myc* and *CCND1*, show opposite tendencies. However, the upstream gene, *SPRY2*, was increased irrespective of V protein/NDV infection and inhibitor treatment. This may infer that V protein/NDV infection can trigger ERK pathway signals not only by phosphorylating ERK1/2, but also by affecting some other targets in this pathway. Further investigations need to determine whether the V protein directly targets ERK1/2 or acts through other host proteins. Overall, our results suggest that the V protein can increase the activity of MEK/ERK pathway through ERK1/2 proteins to benefit viral replication and thus, may be a potential target to control this process.

## 4. Discussion

Burotto et al. reviewed studies and showed that the MAPK/ERK pathway is activated by upstream genomic events and/or activation of multiple signaling events. Furthermore, they concluded that the information coalesced at this important nodal pathway point [29]. The replication cycle of some viruses can stimulate the ERK pathway. For example, the Epstein-Barr virus induces the epithelial-to-mesenchymal transition through ERK-MAPK signaling [30,31]. Coxsackievirus B3 [32], HCV [27] and avian influenza virus [33] also can stimulate the ERK pathway. For NDV infections, using U0126 to suppress ERK activity can downregulate NDV-induced interferon-γ expression. This suggests that NDV may stimulate the ERK pathway in dendritic cells [34]. 

One study reported that the upregulation of the Raf kinase inhibitory protein restricted the replication of the NDV NA-1 strain, while this also repressed the activation of the ERK signaling pathways in the DF-1 cell line [17]. Another report suggested that the susceptibility of the tumor cells to the NDV MTH-68/H strain may be affected by alterations other than those in RAS/ERK signaling in uninfected cells [23]. Moreover, using the NDV MTH-68/H strain, there was no correlation between the IC50 values and the expression of p-ERK in different tumor cells [23]. In our work, we obtained similar results with both NDV strains tested (La Sota and F48E9) in terms of the activation of ERK signaling pathways in HeLa cells.

The ORF45 protein of the rhesus rhadinovirus forms a complex with ERK2 to promote its nuclear accumulation, thereby promoting lytic gammaherpesvirus viral gene expression [35]. In the current study, NDV-induced p-ERK1/2 was also located in the cytoplasm and around the nucleus (Figure 1C). This suggests that p-ERK may affect NDV replication in a similar way to lytic gammaherpesvirus.

The MAPK/ERK signaling pathway is involved in eukaryotic cell proliferation, differentiation, migration, senescence and apoptosis [36]. The regulation of replication by the ERK signaling pathway also has been reported in several viruses and for most viruses reviewed by Takahashi and Suzuki [37], the activation of the ERK pathway was required for virus replication (e.g., influenza A [37], enterovirus 71 [38], yellow fever virus [39], Junín and Tacaribe viruses [40], Japanese encephalitis virus [41], porcine epidemic diarrhea virus [42] and exogenous avian leukosis virus [43]). Both ERK1 and ERK2 were required for the efficient replication of enterovirus 71 [44], but the effect was variable for HCV. Ribavirin can interfere with the ERK and STAT signaling pathways to inhibit HCV replication in human hepatoma Huh7.5.1 cells [45]. In contrast, the overexpression of ERK1 prevented HCV replication in these cells [5]. In our present study, the treatment of HeLa cells with ERK inhibitors suppressed NDV replication, suggesting that the activation of ERK signaling pathways may benefit NDV replication.

Viral infection and replication are complicated processes and it is difficult to ascertain the molecular mechanisms that activate ERK signaling. The studies with ERK inhibitors and agonists indicate that most viral proteins can trigger ERK signaling. For example, the HCV E2 protein can trigger ERK signaling and affect cellular receptor-mediated signaling [46]. The 5A protein of HCV can also mediate ERK signaling [47]. The avian reovirus protein p17 functions as a nucleoporin Tpr suppressor, inactivating the phosphoinositide 3-kinase/Akt/mammalian target of rapamycin and ERK signaling pathways [48]. However, the pseudorabies viral Us2 protein binds to ERK and inhibits the activation of ERK nuclear targets by sequestering cytoplasmic ERK on cellular membranes [49]. Herpes simplex virus 1 virus-encoded Us3 Ser/Thr protein kinase expression in uninfected cells is necessary and sufficient for suppressing ERK activity in the absence of any other virus-encoded gene products [15]. The expression of the varicella-zoster viral ORF12 protein triggers the phosphorylation of ERK1/2 and inhibits MeWo cell apoptosis [50].

The V protein is known to benefit NDV replication [4,5]. We found that the V protein can reduce DF-1 cell apoptosis [51]. However, there were no studies focusing on which NDV protein(s) could trigger ERK signaling. The novel finding reported in Figure 3 is of relevance in the current study as we showed that the V protein triggers the phosphorylation of ERK1/2 and enhances NDV replication in HeLa cells. Further investigation is needed to determine how the V protein triggers the phosphorylation of ERK1/2 and which other viral proteins are involved in the viral replication regulatory mechanism.

## 5. Conclusions

In summary, the results of this study show that the NDV V protein induces the host cell ERK phosphorylation, which subsequently results in enhanced NDV replication. These results suggest that the ERK pathway is exploited as an antiviral defense mechanism, which potentially indicates a new avenue for the development of antiviral strategies and describes a novel mechanism by which the V protein aids viral replication.

## Figures and Tables

**Table 1:** Reagents and primer sequences.

Name	Source or Forward Primer 5′-3′	Identifier or Reverse Primer 5′-3′
Rabbit anti p-ERK1/2 antibody	Cell Signaling Technology	Cat #4370
Mouse anti H3 antibody	Sungene Biotech	Cat #KM9005T
Chicken anti-NDV polyclonal	Immune serum	Prepared in our lab
Goat anti-IgY antibodies (488)	Abcam	Cat #ab150169
Goat anti-mouse antibody (594)	Invitrogen	Cat #SA5-10168
Methyl cellulose	Sigma-Aldrich	Cat #M7027
Hoechst 33342	Sigma-Aldrich	Cat # B2261
Immobilon-P Membrane, PVDF	EMD-Millipore	Ca t# IPVH00010
Clarity™ Western ECL	Bio-Rad	Cat #1705060
1st Strand cDNA Synthesis Kit	Takara	Cat #6210A
2× PCR mixture (qRT-PCR Mix)	Gene Star	Cat #A301-01
C-myc primer	CGTCCTCGGATTCTCTGCTC	GCTGGTGCATTTTCGGTTGT
C-fos primer	AGACCGAGCCCTTTGATGAC	TGGTGTGTTTCACGCACAGA
ATF1 primer	ACCTGGTTCAGCAGTTCAGG	TGGGGCAATGGCAATGTACT
CCND1 primer	CAATGACCCCGCACGATTTC	AAGTTGTTGGGGCTCCTCAG
SPRY2 primer	TCCATAAGCACGGTCAGCTC	GCTTGTCGCAGATCCAGTCT
β-Actin primer	AGACCTGTACGCCAACACAG	TTCTGCATCCTGTCGGCAAT
V (clone)	*GAATTC*ATGGCGACCTTTACAGACGC	CTCGAGTTACTTACCCTCTGTGATATCG
NDV *M* gene	AAGAAGCAAATCGCCCC	ACGCTTCCTAGGCAGAG
NDV-specific reverse-transcription primer	AGGGTTCCCGTTCATTCAG	
